# Arsenic Toxicology: Translating between Experimental Models and Human Pathology

**DOI:** 10.1289/ehp.1103441

**Published:** 2011-06-17

**Authors:** J. Christopher States, Aaron Barchowsky, Iain L. Cartwright, John F. Reichard, Bernard W. Futscher, R. Clark Lantz

**Affiliations:** 1 Department of Pharmacology and Toxicology, University of Louisville, Louisville, Kentucky, USA; 2 Department of Environmental and Occupational Health, University of Pittsburgh, Pittsburgh, Pennsylvania, USA; 3 Department of Molecular Genetics, Biochemistry, and Molecular Biology, and; 4 Department of Environmental Health, University of Cincinnati, Cincinnati, Ohio, USA; 5 Department of Pharmacology and Toxicology, and; 6 Department of Cell Biology and Anatomy, University of Arizona, Tucson, Arizona, USA

**Keywords:** arsenic, biomarkers, dosimetry, epigenetics, gene expression, phenotypic anchoring

## Abstract

Background: Chronic arsenic exposure is a worldwide health problem. How arsenic exposure promotes a variety of diseases is poorly understood, and specific relationships between experimental and human exposures are not established. We propose phenotypic anchoring as a means to unify experimental observations and disease outcomes.

Objectives: We examined the use of phenotypic anchors to translate experimental data to human pathology and investigated research needs for which phenotypic anchors need to be developed.

Methods: During a workshop, we discussed experimental systems investigating arsenic dose/exposure and phenotypic expression relationships and human disease responses to chronic arsenic exposure and identified knowledge gaps. In a literature review, we identified areas where data exist to support phenotypic anchoring of experimental results to pathologies from specific human exposures.

Discussion: Disease outcome is likely dependent on cell-type–specific responses and interaction with individual genetics, other toxicants, and infectious agents. Potential phenotypic anchors include target tissue dosimetry, gene expression and epigenetic profiles, and tissue biomarkers.

Conclusions: Translation to human populations requires more extensive profiling of human samples along with high-quality dosimetry. Anchoring results by gene expression and epigenetic profiling has great promise for data unification. Genetic predisposition of individuals affects disease outcome. Interactions with infectious agents, particularly viruses, may explain some species-specific differences between human pathologies and experimental animal pathologies. Invertebrate systems amenable to genetic manipulation offer potential for elaborating impacts of specific biochemical pathways. Anchoring experimental results to specific human exposures will accelerate understanding  of mechanisms of arsenic-induced human disease.

Arsenic exposure via drinking water affects > 140 million people worldwide and causes cancer and bronchopulmonary, cardiovascular, and metabolic diseases and neuropathies. Various experimental models have been developed to understand how arsenic exposure causes these diverse disease outcomes. Translation of laboratory arsenic toxicology studies to human health is important but is complicated by inexact dose conversion between *in vitro*, murine, and human exposures and species-specific metabolic differences. Here, we discuss issues in dose conversion and potential means to translate findings in selected experimental model systems to an understanding of human arsenic toxicology. Phenotypic anchoring of results from model systems by tissue dosimetry, gene expression and epigenetic mark profiling, and tissue biomarker identification should promote development of a coherent picture of mechanisms of arsenic-induced human disease. We discuss research needs critical to progress in translation of experimental findings. We also highlight a human-specific disease end point and discuss advantages of invertebrate systems to address specific questions in a simpler background with fewer confounding factors.

## Dose and Exposure Conversion

Data collected in human studies often include exposures but not doses. Urine and toenail arsenic are often used as indicators of body burden but are subject to wide individual variation with similar exposures. Dose conversion between human and murine exposures is a complicated issue. Calculating dose requires careful determination of amounts consumed and is rarely reported. Often, consumption estimates are based on data from published studies. However, water consumption can vary greatly in mice and is markedly different in different strains (Bachmanov et al. 2002). Likewise, human exposure data include an estimate of arsenic-contaminated water and/or  food consumption. However, body weights are not systematically collected and differ greatly with study population. Hence, calculation of human dose with individual precision has not been done. Even with reliable dose estimates, dose conversion between the mouse and human is complicated. An estimate based on body surface area may be reliable for many substances (Reagan-Shaw et al. 2008), but arsenic metabolism is strikingly different in rodents and humans (Vahter 1999). For these reasons, anchoring results by induced phenotype may be a more useful approach. A simple anchor might be target tissue arsenic levels. Murine tissue dosimetry can be performed readily, although most data currently available are from mice with high arsenic exposures (Devesa et al. 2006; Gentry et al. 2005). Some human data on tissue, blood, and urine arsenic levels have been correlated with exposures in specific populations. Thus, this approach is limited in that data available are on a population level, but there are no systematic compilations of these correlations on an individual level. Hence, no direct connection between a specific human exposure and a biological arsenic level is available, and research including these measures is needed. Other approaches to determine exposure equivalence by induced phenotype include anchoring by changes in gene expression, epigenetic marks, or tissue remodeling biomarker profiles. These approaches are certainly possible within laboratory models and could readily serve to unify results from experimental systems. However, only very limited data sets are available for human exposures. Thus, there is a great need for research collecting these data from humans who exhibit arsenic-induced disease. These data are critical to translation of experimental results to specific human exposures.

*Transplacental exposures. In utero* exposures to environmental toxicants can have a profound effect on development of chronic adult diseases. Endocrine disruptors are paradigms of developmental toxicants and are linked to diseases as diverse as prostate cancer (Ho et al. 2006) and obesity (Grun and Blumberg 2009). Consequences of *in utero* arsenic exposure in humans are difficult to determine in most cases because exposure is not limited to the *in utero* period but continues into postnatal life. However, a unique situation with a defined period (1958–1971) of arsenic exposure occurred in Antofagasta, Chile (Borgono et al. 1977). This unfortunate incident provides a cohort with a defined period of exposure. Increased incidence of a variety of disease conditions associated with the arsenic exposure was reported shortly after the switch back to low-arsenic water (Borgono et al. 1977). These conditions included increased incidence of bronchopulmonary and cardiovascular diseases, both now clearly linked to chronic arsenic exposure (Argos et al. 2010). Long-term follow-up studies of this cohort revealed high mortality from lung cancer and bronchiectasis in the population exposed *in utero* and during early childhood decades after high exposure ended (Smith et al. 2006). Additionally, the incidence of myocardial infarction in infants whose mothers were exposed during this period (Rosenberg 1974) indicates that *in utero* arsenic exposure could induce cardiovascular disease.

In contrast to the striking results from the Antofagasta population, infant mortality—but not spontaneous abortion—showed dose correlation in a Bangladeshi population (Rahman et al. 2010). This difference may be due to a difference in exposure levels. The water arsenic level in Antofagasta during the high exposure period was approximately 800 μg/L and uniform in the population because there was a single source of water, whereas the Bangladeshi population experienced variable exposures due to multiple sources: 268–2,019 μg/L (median, 390 μg/L) for infant mortality and 249–1,253 μg/L (median, 382 μg/L) for spontaneous abortion study populations. Taken as a whole, *in utero* exposure to high levels of arsenic in drinking water appears to be necessary for obvious adverse effects early in postnatal life. It is likely that lower exposures have a more subtle effect, perhaps contributing to chronic adult diseases.

High arsenic exposure *in utero* affects gene expression in leukocytes from human cord blood (Fry et al. 2007). Gene ontology analysis of altered mRNA expression in arsenic-exposed samples revealed that immune, inflammatory, and stress response categories were affected. Network analyses identified JUNB, interleukin (IL) 8, IL1β, and hypoxia-inducible factor-1α, which are involved in cell cycle regulation, stress response, inflammation, and response to hypoxia, respectively. In addition, nuclear factor-κB was integrated into the subnetworks and also found to be activated in the cord blood of arsenic-exposed infants.

Animal studies indicate that *in utero* arsenic  exposures induce both cancer and atherosclerosis. *In utero* arsenic exposure (42.5 or 85 ppm) induced tumors in C3H mice (Waalkes et al. 2003) and established the first reproducible laboratory animal model of carcinogenesis by inorganic arsenic (iAs) alone. Recent work from this group shows that whole-life exposure at lower levels (6–24 ppm) results in higher tumor incidence (Tokar et al. 2011). Combined with *in vitro* studies showing enhanced proliferation of stem cells, these results led to the hypothesis that cancer induced by *in utero* arsenic exposure is a consequence of arsenic-induced increase in the stem cell population of target tissues (Tokar et al. 2010).

Both *in utero* (Srivastava et al. 2007) and postweaning (Srivastava et al. 2009) exposures to arsenic in drinking water accelerate and exacerbate atherogenesis in the apolipoprotein E-knockout (ApoE^–/–^) mouse model for atherogenesis. These studies showed that atherogenesis was induced by arsenic exposure alone, without the high-fat diet normally used to induce atherosclerosis in this model. The *in utero* arsenic exposure (49 ppm) used in the ApoE^–/–^ experiments produces arsenic levels in livers of the pregnant dams (States JC, unpublished data) similar to those observed in livers of people exposed to high levels of arsenic (200–600 ppb) in drinking water in West Bengal (Guha Mazumder 2001). Data from gene expression analyses show induction of immune, inflammatory, and stress response pathways in livers of 10-week-old ApoE^–/–^ mice exposed to arsenic *in utero* (States JC, unpublished data). These pathways were among the top pathways activated in human cord blood lymphocytes discussed above. Hence, the data suggest that these responses are induced in multiple tissues and may be a common basis from which disease processes emerge. Thus, correlation exists in phenotypic anchors (tissue arsenic levels, altered gene expression) between the higher arsenic exposures in Chile and South Asia and these mouse exposures.

*Arsenic-induced tissue remodeling.* Adverse health effects of chronic arsenic ingestion on the lung include chronic obstructive pulmonary disease, chronic bronchitis, and bronchiectasis. In separate studies in West Bengal and Bangladesh, chronic arsenic exposure reduced lung function (De et al. 2004; Parvez et al. 2008; von Ehrenstein et al. 2005) and increased respiratory disease symptoms (i.e., cough, chest sounds, shortness of breath) and chronic bronchitis (Mazumder et al. 2005; Milton and Rahman 2002). More than 63% of subjects with mean arsenic exposure of 216 ± 211 ppb (compared with 11 ± 20 ppb in controls) displayed increased respiratory complications (Islam et al. 2007). Clearly, high-level arsenic exposure (200–1,000 ppb) causes adverse respiratory effects. However, effects of lower exposures are not known.

Airway remodeling is a hallmark of many respiratory diseases, including emphysema, asthma, idiopathic pulmonary fibrosis, and bronchiectasis (Jeffery 2001; Niimi et al. 2005; Reynolds et al. 2005). Persistent structural changes in tissue develop through a process of injury and dysregulated repair, leading to chronic inflammation and altered extracellular matrix deposition in the airway wall, eventually obstructing airflow. Chronic lung disease phenotypes in populations with high arsenic exposure suggest that extracellular matrix, aberrant cell motility, and wound repair are arsenic targets. Data support this hypothesis because changes in expression and organization of extracellular matrix genes and in expression of mediators and enzymes that control matrix remodeling have been observed consistently in a wide range of model systems.

Expression of a large number of extracellular matrix genes was altered in adult male C57Bl/6 mice exposed to either 10 or 50 ppb arsenic in their drinking water for up to 8 weeks (Lantz and Hays 2006). These alterations included suppression of several collagen, elastin, and fibronectin isoforms. In addition, mRNA for matrix metalloproteinase-9 (MMP-9), a matrix degradation enzyme, was induced. Disorganization and expansion of elastin and collagen after 8-week 50 ppb arsenic exposure were observed around pulmonary airways and blood vessels. Arsenic-induced changes in adult animals also occurred in the extravascular matrix of small cardiac arteries (Hays et al. 2008).

Matrix is also critical for cell migration, wound repair, and remodeling after injury. Pathway analysis using gene and protein expression data from multiple model systems suggests that wound repair and cell motility are two of the more probable processes affected by arsenic exposure (Lantz and Hays 2006; Lantz et al. 2007, 2009; Petrick et al. 2009). Arsenic increased time to close a scratch wound in confluent human airway epithelial cells. This increased closure time (reduced wound repair) was associated with increased expression and activity of MMP-9. Arsenic, even in the absence of the wounding, induced significant production of MMP-9, and inhibition of MMP-9 partially restored repair. Inhibition of repair also occurs in an animal model. Animals exposed to arsenic had less capacity to repair naphthalene-induced airway injury (Lantz RC, unpublished data).

During fetal and early postnatal lung development, extracellular matrix gene expression is necessary for proper development of lung and blood vessels. In the highly exposed Antofagasta population, *in utero* and early postnatal exposure (~ 800 ppb) increased risk of chronic obstructive pulmonary disease and bronchiectasis (Smith et al. 2006). After *in utero* and early postnatal exposure in mice (≤ 100 ppb), lung collagen type 1 α2 (*Col1a2*), *Col3a1*, and elastin mRNA expression increased and exhibited both developmental time and exposure dependence (Lantz et al. 2009). Changes in matrix protein expression may result from arsenic interaction with normal developmental processes. However, whole-lung collagen and elastin levels were not significantly altered. Increased mRNA expression could be a compensatory response. For example, arsenic-induced increases in MMP-9 during early postnatal periods, as seen in a mouse model, would degrade matrix, requiring increased mRNA expression to maintain appropriate protein levels.

Although whole-lung levels of matrix proteins were unchanged, regional decreases in total collagen in adventitia around airways were seen in 28-day-old mice exposed to arsenic during development (Lantz et al. 2009). Localized decreases in collagen were associated with increased levels of smooth muscle around airways and alterations in pulmonary function. Understanding mechanisms for localized arsenic effects requires research.

Of critical importance is whether changes seen in model systems replicate events in human populations. Levels of MMP-9 and its inhibitor, TIMP-1 (tissue inhibitor of metalloproteinase-1), determined in populations with low exposures to arsenic (< 20 ppb) through drinking water showed that the MMP-9:TIMP-1 ratio in induced sputum was positively associated with total urinary arsenic (Josyula et al. 2006). Although the underlying mechanism is different from that in model systems (increased ratios were due predominantly to TIMP-1 decreases in humans), the underlying effect—increased degradation of matrix—is the same.

Thus, ingested arsenic alters matrix and matrix-associated proteins in a number of model systems and in humans. Evaluation of arsenic-induced phenotypic alterations, including lung function, lower respiratory infections (predicted from model systems), and changes in mediators affecting matrix deposition, is needed, especially in children. Changes in matrix deposition may be a source of useful tissue biomarkers for phenotypic anchoring.

*Arsenic-induced vascular disease in adult animal models.* Arsenic exposure is strongly associated with increased cardiovascular disease risk (States et al. 2009). High exposures cause occlusive arteriosclerosis, such as blackfoot disease seen in Taiwan (Tseng 2008) and coronary occlusion in infants in Chile (Rosenberg 1974). Many studies have found increased cardiovascular disease risk with more modest exposures (10–100 ppb). In the United States, mortalities from vascular diseases were increased in counties where arsenic levels were > 20 ppb relative to those with < 10 ppb (Engel and Smith 1994). Diseases associated with these lower exposures include coronary artery and ischemic heart disease, carotid atherosclerosis, microcirculatory defects, and prolonged QT intervals (Medrano et al. 2010; Tseng 2008; Wang et al. 2009). Arsenic may increase associated vascular disease risk factors, such as systolic hypertension (Chen et al. 2009; Tseng 2008) and diabetes (Navas-Acien et al. 2009). Increased systolic hypertension (Chen et al. 2009) is consistent with direct stimulatory effects of arsenic on vascular smooth muscle (Soucy et al. 2004) and decreased vasorelaxation (Srivastava et al. 2007, 2009). Nutritional (Chen et al. 2009), metabolic (Mazumder et al. 2005; Navas-Acien et al. 2005), and genetic susceptibilities (States et al. 2009) to cardiovascular pathologies caused by arsenic implicate enhanced oxidant signaling as a primary mode of action. This appears as endothelial cell dysfunction and metabolic dysregulation from loss of nitric oxide or gain of oxidant signaling (States et al. 2009).

Mice may be as sensitive as or more sensitive than humans to vascular pathologies caused by low to moderate arsenic exposures. Angiogenesis, tumor angiogenesis, and liver sinusoidal vessel remodeling occur in C57BL/6 mice exposed for 2–5 weeks to 1–10 ppb arsenic (Soucy et al. 2003, 2005; Straub et al. 2008). Mouse models reproduce the atherogenic effects of arsenic after *in utero* (Srivastava et al. 2007) or adult arsenic exposures (Bunderson et al. 2004; Srivastava et al. 2009). In the mouse heart, arsenic caused perivascular fibrosis (Hays et al. 2008) and increased expression of matrix remodeling proteins (e.g., Serpine1 and MMP-9) (Soucy et al. 2005). At higher exposures, progressive loss of myocardial microvessels (Soucy et al. 2005) and cardiomyopathy (Li et al. 2002) occurred. In the developing chicken heart, arsenic affects epithelial to mesenchymal transitions necessary for valves to develop (Lencinas et al. 2010). Arsenic causes liver steatosis, fibrosis, and portal hypertension in humans (Mazumder 2005) that may predispose individuals to risk of systemic atherosclerosis and metabolic disease (Targher et al. 2010). Arsenic causes mouse liver sinusoidal endothelial cell (LSEC) capillarization and periportal vessel hyperplasia (Straub et al. 2007, 2008) that resemble similar pathology seen in infants who died from *in utero* or perinatal arsenic exposures (Rosenberg 1974). As in humans, studies in rabbit models (Pi et al. 2003) and mouse models (Bunderson et al. 2004; Straub et al. 2008) implicated nitric oxide loss and increased oxidant signaling in promoting endothelial cell dysfunction and pathogenic phenotypic change. Thus, animal models recapitulate pathogenic end points that are relevant to arsenic-induced human cardiovascular diseases, and these end points provide phenotypic anchors for systematic investigation of pathogenic mechanisms.

Phenotypic anchors of vessel remodeling and vessel cell-to-matrix interactions involved in remodeling reveal critical signaling pathways underlying the etiology of arsenic-related vascular diseases. Matrix interactions are critical for maintaining vessel integrity, wall cell phenotype, and functional signaling. In a model of epithelial to mesenchymal transition in heart valve development, transcriptomic analysis revealed 382 genes that were responsive to 25 ppb arsenic (Lencinas et al. 2010). Pathway analysis identified clusters of responsive genes involved in cytoskeletal regulation, matrix deposition, and cell adhesion, as well as in stabilizing an endothelial cell phenotype (Lencinas et al. 2010). The cluster of cytoskeletal-regulating genes included GTPases (*Rac1* and similar members of the RhoA GTPase family) known to be activated by arsenic in vascular dysfunction (Qian et al. 2005; Smith et al. 2001; Straub et al. 2007, 2008) and inflammation (Lemarie et al. 2008). *In vivo*, arsenic exposure results in membrane localization of Rac1 in capillarized LSEC (Straub et al. 2007, 2008). In *ex vivo* studies, arsenic-induced LSEC capillarization was prevented by inhibiting Rac1 activity (Straub et al. 2007, 2008). Rac1 also is highly expressed in skin tumors induced by arsenic plus phorbol ester in Tg.AC mice (Waalkes et al. 2008).

The Rac1 signaling program mediates arsenic-induced generation of reactive oxygen species that are second messengers for its pathogenic effects. Rac1 is an essential subunit of Nox2-type NAPDH oxidase, and this oxidase is required for arsenic-stimulated large-vessel endothelial and LSEC oxidant production (Smith et al. 2001; Straub et al. 2008). Arsenic does not capillarize LSEC in mice lacking this oxidase (Straub et al. 2008). This finding was the first *in vivo* demonstration of a role for NADPH oxidase in arsenic action and the first demonstration that the activation of the oxidase promotes LSEC capillarization. In a recent study Ghatak et al. (2010) confirmed that NADPH oxidase activity is central to arsenic-induced liver fibrosis. Further, chronic activation of Rac1 and Nox2-type NADPH oxidases are longitudinal risk factors for vascular disease and hypertension (Lee and Griendling 2008). Gain-of-function polymorphisms in oxidase subunit genes are associated with cardiovascular disease in general (San et al. 2008) and with arsenic-induced disease (States et al. 2009).

There is a significant knowledge gap in understanding how phenotypic change in individual cell types relates to pathogenic vascular remodeling and function. Arsenic-induced LSEC capillarization limits the removal of lipoproteins, lipids, and waste proteins from the circulation and alters normal liver lipid metabolism (Straub et al. 2008). In addition, zonal distribution of hepatocyte lipid deposition changes from being exclusively within hepatocytes surrounding the central veins (zone 3) to spreading into hepatocytes surrounding the portal veins (zone 1). These effects may translate to both liver and systemic vascular diseases (Targher et al. 2010). Arsenic-induced change in liver cell phenotype and underlying cell matrix appears to alter basic liver structure, function, and metabolism. However, full investigation of the LSEC responses is hindered by the overwhelming mass of hepatocytes masking these responses. Preliminary evaluation of total mouse liver mRNA, microRNA, and proteome responses to lower level arsenic exposures revealed modest changes (Straub et al. 2009). This modest effect is expected because there is little observable arsenic-induced change in the hepatocytes. Examination of primary LSEC exposed to arsenic *ex vivo*, however, demonstrated much greater responses that supported the pathogenic *in vivo* effects (e.g., decreased expression of the scavenger receptor stabilin-2). The challenges are to determine whether LSEC-specific or vascular-cell–specific changes can provide markers for arsenic-induced pathogenesis and whether preventing arsenic effects in LSECs or vascular cells prevents systemic pathogenesis. Similarly, there is a need to understand how arsenic-induced change in microvascular phenotype affects organ function, such as in the liver, or systemic metabolic changes that promote cardiovascular and metabolic diseases.

*Epigenetic effects of arsenic exposure.* The disruption of normal epigenetic control can participate in the etiology of complex human diseases, including psychiatric disorders, cardiovascular disease, diabetes, and cancer. In cancer, pathologic disruption of the normal epigenetic state of a cell can be caused by diverse mediators and mechanisms, including environmental agents, stresses, and cues. Accumulating evidence indicates that arsenic is an environmental toxicant that can mediate epigenetic changes (Reichard et al. 2007; Ren et al. 2011b). Thus, epigenetic control mechanisms are a nexus of gene–environment interactions that link cellular responses to arsenic exposure. DNA and histone modification enzymes and the cellular pathways that input signals to them represent potential targets for disruption leading to an altered epigenetic state and phenotype.

Recent work links arsenic exposure to epigenetic state disruption and progression of the diseased state. During carcinogenesis, arsenic exposure induces global DNA hypomethylation with hypomethylation frequently found in repetitive elements, although DNA demethylation of some gene regulatory regions also occurs (Chen et al. 2004; Jensen et al. 2009a; Reichard et al. 2007). The functional consequences of this DNA hypomethylation remain unclear but may involve inappropriate gene activation or altered chromatin structures. Because arsenicals inhibit activity of DNA methyltransferases DNMT1 and DNMT3a (Reichard et al. 2007), this effect may contribute to overall decreased levels of DNA methylation. However, it may be only one of multiple factors contributing to arsenical-induced epigenetic change, because arsenicals also mediate a coincident DNA hypermethylation of CpG island gene promoters, as well as changes in histone posttranslational modifications.

Aberrant DNA hypermethylation of CpG island gene promoters is functionally linked to inappropriate transcriptional silencing, and disease progression. This epigenetic lesion has been found in multiple human cell models of arsenical-induced malignant transformation (Cui et al. 2006; Jensen et al. 2009a). In one example, both arsenite and monomethylarsonous acid (MMA^III^) induced malignant transformation of an immortalized urothelial cell line model of human bladder cancer (UROtsa) (Bredfeldt et al. 2006; Sens et al. 2004). In this model, arsenite and MMA^III^ each induced hundreds of DNA methylation changes across the genome, with a striking overlap in genes targeted by these similar but chemically distinct arsenicals. These results suggest that different forms of arsenic may act similarly in their ability to perturb the epigenetic landscape. For example, in the UROtsa model, both MMA^III^ and arsenite induced DNA hypermethylation-associated gene silencing of *DBC1* (deleted in bladder cancer 1) and *G0S2* (G0/G1 switch regulatory protein 2) (Jensen et al. 2008, 2009a). Interestingly, both of these genes display tumor suppressor function and become aberrantly methylated and transcriptionally silenced in clinical bladder cancer (Chang et al. 2010; Habuchi et al. 1998; Hoque et al. 2006; Izumi et al. 2005; Kusakabe et al. 2010; Welch et al. 2009), suggesting that *in vitro* models of arsenical-induced malignant transformation may accurately reflect epigenetic events that occur in clinical disease. The human relevance of these *in vitro* studies is further suggested by recent human population-based studies that found a connection between arsenic exposure and epigenetic dysfunction in bladder cancer (Marsit et al. 2006).

Many of the arsenic-mediated epigenetic gene-silencing events linked to gene promoter DNA hypermethylation were also accompanied by changes in the histone code in these same regions, specifically hypoacetylation of histones H3 and H4 (e.g., Jensen et al. 2009a). The temporal order of and mechanisms involved in this multifaceted epigenetic reprogramming are not clear. The epigenetic state change may result from a new epigenetic program being enacted by arsenical-driven alterations in cell signaling inputs. Alternatively, arsenicals may act on multiple epigenetic modifier enzymes to short-circuit the epigenetic program. Indeed, changes in both histone phosphorylation and histone methylation that appear independent of DNA methylation changes occur after arsenical exposure (Jensen et al. 2009b; Zhou et al. 2008). Taken together, these results indicate that arsenicals likely disrupt multiple epigenetic pathways.

Epidemiological studies of Chilean populations show an arsenic-related increase in lung and bladder cancer mortality, as well as a long latency between the time of major arsenic exposure and increased disease rates (e.g., Marshall et al. 2007). The long latency suggests that arsenicals may damage the epigenomic integrity of progenitor or stem cell populations and that the expanded populations arising from these progenitors retain the epigenetic changes. This type of epigenetic initiation event is consistent with the first step in the recently proposed epigenetic progenitor theory of carcinogenesis (Feinberg et al. 2006). Specifically, we predict that arsenicals induce changes in the epigenetic terrain of progenitor cells that are faithfully inherited from cell generations, even after removal of the initiating toxicant. Thus, arsenicals may act as epimutagens—agents capable of altering the epigenome of cell populations, resulting in changes in gene expression and phenotypic shifts. This long-term epigenetic damage may remain silent until other critical events occur (e.g., loss of *p53*, immortalization), at which time the arsenical-induced epigenetic changes may be phenotypically “unmasked” and help drive evolution of the malignant phenotype (e.g., suppression of tumor suppressor genes). The precise mechanisms responsible for arsenic’s disruption of a cell’s epigenetic state are being elucidated and will be critical for a full understanding of arsenical action. Research profiling epigenetic changes in human tissues is needed to validate the epigenetic changes observed *in vitro*.

*Cutaneous effects of arsenic and human papilloma viruses.* In humans, skin is the most sensitive target organ for chronic arsenic exposure (Yoshida et al. 2004). Even at low-level exposures, arsenic increases risks for pigmentation changes (melanosis), hyperkeratosis, Bowen’s disease, and nonmelanoma skin cancer (NMSC) (Agency for Toxic Substances and Disease Registry 2007; Chen et al. 2009). Although chronic arsenic exposure is causally linked with skin disease, cutaneous arsenicosis is solely a human disorder for reasons that remain unknown (Rossman et al. 2002). However, human-specific hyperkeratosis may be linked to enhanced viral infection and immune suppression observed in laboratory studies.

One possible explanation for the human specificity of the effect of arsenic on skin is an interaction with a viral skin pathogen. Arsenic exposures inhibit immune function, at least in part by inhibiting immune surveillance of dendritic cells and CD4 cell activation (Lantz et al. 1994; Liao et al. 2009). By compromising immune function, arsenic impairs the immune response to viruses. This effect has been demonstrated for influenza A, for which arsenic exposure elevates viral titers and increases morbidity (Kozul et al. 2009; Yu et al. 2006). Similarly, it has been known for more than a century that arsenic exposure can reactivate latent herpes infections (Au and Kwong 2005; Lanska 2004). Likewise, human papillomavirus (HPV), a human-specific pathogen, shares several clinical features with arsenicosis and may contribute to arsenical skin disease. Cutaneous HPV establishes infection by evading detection by skin dendritic cells (Langerhans cells). Therefore, it is reasonable that immune inhibition by arsenic could unmask preexisting infections or impair the immunologic response to new exposures (Frazer et al. 1999).

Most individuals are exposed to dermal HPV during their lifetimes (Pfister 2003). In fact, many individuals have antibodies against HPV, thereby demonstrating prior exposure (Masini et al. 2003). Such exposures may be of little consequence for individuals with normal immune function; however, individuals with impaired immune function are at significantly increased risk of HPV infection and NMSC. Patients with epidermodysplasia verruciformis have an immune defect that prevents recognition of HPV, resulting in severe skin infection and a 90% increase in NMSC risk (Pfister 2003). Likewise, immunosuppressive therapy increases the risk of skin warts and premalignant actinic keratoses 2-fold and risk of squamous cell carcinoma 150-fold (Shamanin et al. 1996; Stockfleth et al. 2004). Thus, arsenic-induced immune suppression may increase HPV infectivity.

Only a handful of studies have investigated the occurrence of HPV infection in dermal arsenicosis. Ninety NMSC patients recruited from an arsenic-endemic region of Mexico were evaluated for the serological presence of HPV-16–reactive antibodies (Rosales-Castillo et al. 2004). The odds ratios for NMSC in patients with a positive history for high arsenic exposure or the presence of antibodies against HPV were 4.53 and 9.04, respectively. This risk increased to 16.5 when both high-level arsenic exposure and HPV were present (Rosales-Castillo et al. 2004). Although it has not been systematically investigated, several case studies have directly detected HPV infection in arsenical skin lesions. HPV types 16 and 41 have been detected in squamous cell carcinomas taken from arsenic-exposed patients (Grimmel et al. 1988; Neumann et al. 1987), and HPV-23 was identified in multiple hyperkeratotic papules from a single patient (Gerdsen et al. 2000). Somewhat in contrast with these findings, Ratnam et al. (1992) detected HPV in only 2 of 33 arsenical keratoses isolated from four patients. The differences among these findings are not surprising given the small study size, the > 100 types of HPV, and the technical challenge associated with broadly detecting cutaneous HPV types (Dang et al. 2006; Vasiljevic et al. 2007).

In addition to arsenic’s effect on immune function, arsenic may promote integration of HPV DNA into the genome of keratinocytes, the process underlying HPV-mediated neoplasia (Jones and Wells 2006). Damage to episomal HPV DNA, such as that caused by oxidative stress, is a critical step triggering genomic integration of the virus and expression of genes that promote keratinocyte proliferation and inhibit differentiation (Jones and Wells 2006). By promoting integration, arsenic may enhance the tumorigenicity of HPV (Germolec et al. 1997; Milner 1969; Rossman 1998). Together, HPV and low-concentration arsenic may target epidermal stem cells to promote keratinocyte proliferation and inhibit normal differentiation (Egawa 2003; Liu et al. 2010). Although the effect of arsenic on HPV-infected cells is unknown, preliminary data suggest that arsenic increases cell division and delays differentiation of HPV-infected keratinocytes in organotypic skin cultures, leading to delayed differentiation, increased suprabasal cell division, and suprabasal skin thickening (Reichard JF, unpublished data). Clearly, more research on arsenic enhancement of viral infections in both animals and humans is needed.

*Metabolism, genetics, and model systems.* Human dose dependence for any arsenic-linked phenotypic outcome depends on multiple critical factors, such as intracellular chemical transformation, tissue distribution, reactivity, and efflux (Thomas 2007). Each can be affected by individual genetic variability, so departures from the “norm” in dose responsiveness and outcome often occur. Use of genetically manipulable models can undoubtedly enhance our understanding of these processes and their importance to toxicity mechanisms.

The methylated derivatives monomethylarsonic acid (MMA^V^) and dimethylarsinic acid (DMA^V^) were believed to be detoxified metabolites (Vahter 1999). However, detection of the methylated As^III^ (+3 oxidation state) species in urine (Le et al. 2000) altered perceptions because MMA^III^ is significantly more toxic than either iAs or the other metabolites (Petrick et al. 2000; Styblo et al. 2000). Some 10–20% of urinary metabolite in humans is MMA [much higher than for most mammals (Vahter 2002)]; the expectation that a portion of this is MMA^III^ might account for higher human susceptibility to pathologic outcomes compared with rodents. Studies of arsenic-exposed populations link urinary MMA levels and individual susceptibility to a range of arsenic-related pathologies (Smith and Steinmaus 2009). The genetic contribution to this association is important, with data suggesting that several pathways might contribute to differential MMA levels (e.g., uptake, one-carbon metabolism, speciation, efflux). The *S*-adenosylmethionine–dependent enzyme arsenic (+3 oxidation state) methyltransferase (AS3MT) is capable of transforming iAs to produce MMA and DMA species of both +3 and +5 oxidation states (Li et al. 2005; Thomas et al. 2004). Certain intronic and extragenic *AS3MT* polymorphisms (along with more extended local haplotypes) are associated with higher DMA:MMA ratios (Gomez-Rubio et al. 2010; Schlawicke et al. 2009), whereas the exon 9 polymorphism M287T is associated with higher urinary levels of MMA (Hernandez and Marcos 2008). Recently, this M287T allele was associated with both elevated damage to DNA (Sampayo-Reyes et al. 2010) and enhanced premalignant skin lesions (Valenzuela et al. 2009), suggesting a mechanistic connection to higher MMA levels. More detailed study of the catalytic properties of AS3MT alleles and their response to input from other intersecting pathways (e.g., one-carbon metabolism, redox environment, feedback inhibition) is required.

In the larger context, more insightful studies into the mechanisms and consequences of arsenic uptake, speciation, distribution, retention, and efflux *in vivo* are necessary. Reports on metabolite-specific transport into and out of cells (Drobna et al. 2010; Liu et al. 2006), as well as mouse studies on organ-specific distribution, retention, and excretion of specific metabolites (Kenyon et al. 2008), have appeared. MMA species can accumulate in cells (perhaps owing to their reactivity), whereas DMA is readily exported. More genetically amenable models are now available for study. Arsenite-fed *AS3MT*-knockout mice produced low levels of methylated metabolites but accumulated high levels of iAs (up to 20-fold higher than wild-type mice) in various tissues (Drobna et al. 2009; Hughes et al. 2010), supporting methylation as a key pathway for arsenic elimination. Such iAs accumulation led to early death (Yokohira et al. 2010).

Organisms such as *Drosophila* and yeast are simpler eukaryotes that have genetic advantages and few confounders. These organisms provide experimentally accessible models capable of rapidly generating fresh insight and testable hypotheses. Thus, *Drosophila* lacks a homolog of *AS3MT*, but introducing the human *AS3MT* gene allows both MMA and DMA species to be produced. Arsenite-fed transgenic flies show important dose-dependent differential effects of these species *in vivo* compared with the wild-type, with significantly impaired chromosomal stability at 9 ppm but enhanced viability at an acute exposure of > 60 ppm owing to reduced arsenic accumulation (Muñiz Ortiz et al. 2011). The data integrate the idea that methylated arsenicals are more damaging to macromolecules yet are more readily eliminated and that iAs dose makes all the difference to phenotypic consequence. Importantly, the quantitative consequences of other human *AS3MT* alleles (e.g., M287T) can be tested readily in this system. The availability of a transcriptome-wide RNA interference-based gene knockdown system in *Drosophila* should provide novel screens that identify pathways intersected by such metabolites. Complementary approaches already initiated in yeast using a gene deletion library have identified novel pathways pertaining to arsenite methylation and histone H4 methylation that are relevant in human cells (Jo et al. 2009; Ren et al. 2011a).

## Disease Outcome Dependence on Interaction with Genetics and Other Environmental Factors

Humans exposed to arsenic do not all succumb to a single disease. Some develop cancer,  whereas others develop cardiovascular disease or neuropathies. The reason for the different responses to similar exposures is unclear. A hint is apparent in the differential response of different strains of mice to similar *in utero* arsenic exposures. C3H, CD, or Tg.AC mice develop earlier and more severe cancer (Tokar et al. 2011; Waalkes et al. 2003, 2008), whereas ApoE^–/–^ mice develop earlier and more severe atherosclerosis (Srivastava et al. 2007, 2009). These responses are clearly linked to the disease predisposition of the mice, and this disposition appears to be aggravated by the arsenic exposure. Thus, arsenic interaction with the genetic background of the organism determines the disease outcome in these models.  In humans, disease outcome also is likely dependent on interaction with other exposures in addition to individual genetic predisposition. Immunosuppression by arsenic exposure may increase susceptibility to infectious agents (Kozul et al. 2009). Thus, increased sensitivity to viral infections could increase oncogenesis if the individual is exposed to oncogenic viruses such as HPV. Chronic arsenic exposure causes hyperreactivity to lipopolysaccharide (Arteel et al. 2008), suggesting that aggravated inflammatory responses to bacterial infections or even to nonpathogenic exposures could be aggravated. Hence, arsenic exposure may prime the system for exaggerated response to a second hit that could be a biological or physical agent, diet, or altered metabolism encoded by individual genetics. Thus, more studies both of human genetics and disease outcome and of structure/function relationships of polymorphic genes involved in arsenic metabolism are needed.

## Conclusions

Chronic arsenic exposure, mostly via contaminated drinking water, causes a multitude of diseases. It is unclear what governs the specific pathology induced in any given individual. However, genetic susceptibility to a particular disease and interaction with other environmental factors play major roles in determining disease outcome of arsenic exposure. Anchoring of experimental models for arsenic toxicology to specific human exposures is essential to gaining a mechanistic understanding of how arsenic exposure leads to specific human pathologies. Global gene expression profiling, epigenetic mapping, and markers of tissue remodeling offer promise as phenotypic anchors. Full development of anchors requires extensive research to profile gene expression, to map epigenetic marks, and to identify biomarkers in target, or surrogate, tissues in arsenic-exposed populations. Human research that includes dosimetry would have greatest impact.
